# *Moringa oleifera* leaf extracts protect BMSC osteogenic induction following peroxidative damage by activating the PI3K/Akt/Foxo1 pathway

**DOI:** 10.1186/s13018-021-02284-x

**Published:** 2021-02-20

**Authors:** Meiling Liu, Haifeng Ding, Hongzhi Wang, Manfeng Wang, Xiaowei Wu, Lu Gan, Luyang Cheng, Xianglu Li

**Affiliations:** Department of Geriatrics, Second Affiliated Hospital of Harbin Medical University, 246 Xuefu Road, Nangang District, Harbin, 150086 China

**Keywords:** *Moringa oleifera* leaf extracts, Bone marrow mesenchymal stem cells, Osteogenic induction, Hydrogen peroxide, PI3K/Akt pathway, Foxo1

## Abstract

**Objective:**

We aimed to investigate the therapeutic effects of *Moringa oleifera* leaf extracts on osteogenic induction of rat bone marrow mesenchymal stem cells (BMSCs) following peroxidative damage and to explore the underlying mechanisms.

**Methods:**

Conditioned medium was used to induce osteogenic differentiation of BMSCs, which were treated with H_2_O_2_, *Moringa oleifera* leaf extracts-containing serum, or the phosphatidyl inositol-3 kinase (PI3K) inhibitor wortmannin, alone or in combination. Cell viability was measured using the MTT assay. Cell cycle was assayed using flow cytometry. Expression levels of Akt, phosphorylated (p)Akt, Foxo1, and cleaved caspase-3 were analyzed using western blot analysis. The mRNA levels of osteogenesis-associated genes, including alkaline phosphatase (ALP), collagen І, osteopontin (OPN), and Runx2, were detected using qRT-PCR. Reactive oxygen species (ROS) and malondialdehyde (MDA) levels, as well as superoxide dismutase (SOD), glutathione peroxidase (GSH-PX), and ALP activity were detected using commercially available kits. Osteogenic differentiation capability was determined using alizarin red staining.

**Results:**

During osteogenic induction of rat BMSCs, H_2_O_2_ reduced cell viability and proliferation, inhibited osteogenesis, increased ROS and MDA levels, and decreased SOD and GSH-PX activity. H_2_O_2_ significantly reduced pAkt and Foxo1 expression, and increased cleaved caspase-3 levels in BMSCs. Additional treatments with *Moringa oleifera* leaf extracts partially reversed the H_2_O_2_-induced changes. Wortmannin partially attenuated the effects of *Moringa oleifera* leaf extracts on protein expression of Foxo1, pAkt, and cleaved caspase-3, as well as mRNA levels of osteogenesis-associated genes.

**Conclusion:**

*Moringa oleifera* leaf extracts ameliorate peroxidative damage and enhance osteogenic induction of rat BMSCs by activating the PI3K/Akt/Foxo1 pathway.

## Introduction

Aging can cause peroxidative damage, which can lead to decreased bone mass and osteoporosis [1], which is closely related to frailty [2], increasing the risk of negative events such as disability, paralysis, and tumble [3]. Osteoporosis is a disease caused by multiple pathogenic factors [4, 5], and severe osteoporosis represents a disease of high mortality and morbidity [6]. Individuals with a high probability of osteoporotic fractures present a very significant disease burden to society, and this burden is set to increase markedly in the future [7]. At present, clinical treatment for osteoporosis is carried out from a single path, but with little efficacy and adverse reactions.

Bone marrow mesenchymal stem cells (BMSCs) are a subgroup of cells with multi-differentiation potential, and their directed differentiation is a complex process involving multiple cellular pathways. BMSCs are defined by their phenotypic expression of CD44, CD90, and CD105, and absence of expression of hematopoietic markers such as CD45, CD34, and CD14 [8]. BMSCs with multi-differentiation properties are currently used to treat a variety of diseases, such as type 1 diabetes, inflammatory bowel disease, and cardiac disease [9–11]. Several studies have demonstrated that BMSCs can be induced to differentiate into bone cells under specific induction conditions [12, 13]. Recently, infusion of BMSCs has been considered a promising strategy to treat osteoporosis. For example, studies in animal models have revealed that both allogeneic and autologous BMSC transplantation is applicable in the treatment of osteoporosis [14]. Accumulating evidence has confirmed that transplantation of BMSCs can promote formation of new bones, improve bone quality, and prevent osteoporosis-associated bone fractures [15–17].

*Moringa oleifera* is an angiosperm plant, and various parts of the plant have been utilized throughout history as food and medicine [18]. In recent years, it has been found that *Moringa oleifera* has good anti-oxidative, hypoglycemic, hypolipidemic, anti-tumor, and anti-fungal activities [19–24]. Studies have confirmed that the Moringa flower extract has the highest antioxidant capacity, followed by the Moringa leaf extract [25]. Remarkably, *Moringa oleifera* leaves have a positive preventive and therapeutic effect on various chronic diseases [26]. Studies have also shown that the high nutrient content and various biological activities of *Moringa oleifera* can improve the oxidative state of postmenopausal women [27], but there is no relevant report on its effects on ameliorating osteoporosis.

In this study, we established an in vitro peroxidative damage model of primary rat BMSCs using H_2_O_2_. *Moringa oleifera* leaf extracts containing serum were used to test the protective effects on BMSCs challenged with peroxidative damage during osteogenic differentiation, and we explored the possible cellular and molecular mechanisms underlying the protective properties of *Moringa oleifera* by using wortmannin (an inhibitor of phosphoinositide-3 kinase) [28].

## Materials and methods

### BMSC cell culture

Primary cultures of rat BMSCs were prepared from eight Sprague-Dawley rats aged 3 to 4 weeks weighing 100 ± 10 g. Male rats maintained in specific-pathogen-free (SPF) conditions were provided by the Animal Experimental Center of the Second Affiliated Hospital of Harbin Medical University, Harbin, China. For the experiments involving in vitro osteogenic differentiation of BMSCs, rats were obtained from the Animal Experimental Center and sacrificed by cervical dislocation after being euthanized with CO_2_ inhalation. This study was approved by the Institutional Animal Care and Use Committee of Harbin Medical University.

After CO_2_ euthanasia, rats were sacrificed by cervical dislocation and soaked in 75% ethanol for 10 min. The bilateral femur and tibia of rats were removed under aseptic conditions. The periosteum and muscle tissue were dissected, and the femur and tibia were washed with phosphate buffered saline (PBS) three times. Next, the medullary cavity was cut and washed repeatedly with DMEM/F12 medium (Hyclone; Logan City, UT, USA) containing 10% fetal bovine serum (Biological Industries; Beit Haemek, Israel). After centrifugation at 1200 rpm for 5 min, the bone marrow cells in the pellets were inoculated into cell culture flasks with complete DMEM/F12 medium and cultured in a humidified atmosphere with 5% CO_2_ at 37 °C. The culture medium was changed for the first time in 48~72 h, and then changed every 2~3 days. When reaching 80% confluence, the adherent cells were passaged after trypsin digestion, and third generation BMSCs (P3 cells) at the logarithmic growth phase were used for subsequent experiments.

To induce commitment toward an osteogenic lineage, P3 cells were cultured in osteogenic differentiation induction (OS) medium, which was comprised of DMEM/F12 culture medium supplemented with fetal bovine serum (10%), 50 μg/ml vitamin C, 10 mmol/L β-glycerophosphate, and 0.1 μmol/L dexamethasone (Solarbio; Beijing, China), as previously reported [29].

### Immunofluorescence analysis of BMSCs

BMSCs at the third passage were fixed with 4% paraformaldehyde for 15 min and blocked with 5% bovine serum albumin (BSA) in TBST (Tris-buffered saline, 0.1% Tween 20). Cells were probed with primary antibodies (1:100 dilution) against CD44, CD90, CD31, and CD34 (Absin, Shanghai, China) overnight at 4 °C. Bound antibodies were detected with Cy3-conjugated goat anti-rabbit IgG (1:400 dilution; Absin, Shanghai, China) and FITC-conjugated goat anti-mouse IgG (1:100 dilution, Zhongsu Jinqiao, Beijing, China), followed by a nuclear staining with 4′,6-diamidino-2-phenylindole (DAPI). Cells stained with secondary antibody only were considered negative controls. Fluorescent signals were detected using a fluorescence microscope (Nikon Eclipse Ti, Nikon, Japan).

### Cell treatment and grouping

The P3 cells cultured in OS medium were treated with H_2_O_2_ (50 μmol/L to 200 μmol/L) or 0.5 μmol/L wortmannin (phosphatidyl inositol-3 kinase (PI3K) inhibitor; Solarbio; Beijing, China), as indicated in each experimental design. H_2_O_2_ (100 μmol/L) was selected to test the impacts of Moringa leaf-containing serum (10%). The P3 cells were divided into the following groups: Table 1

**Table 1:** The experimental groups and treatments

Group	Medium	Treatment
Control	Osteogenic differentiation medium	No
MO	Osteogenic differentiation medium + 10% Moringa leaf-containing serum	No
OS + H_2_O_2_	Osteogenic differentiation medium	100 μmol/L H_2_O_2_
MO + H_2_O_2_	Osteogenic differentiation medium + 10% Moringa leaf-containing serum	100 μmol/L of H_2_O_2_
MO + H_2_O_2_ + Wor	Osteogenic differentiation medium + 10% Moringa leaf-containing serum	100 μmol/L H_2_O_2_ and 0.5 μmol/L wortmannin
MO + H_2_O_2_ + DMSO	Osteogenic differentiation medium + 10% Moringa leaf-containing serum	100 μmol/L H_2_O_2_, 0.5 μmol/L of wortmannin and DMSO (its dosage was the same as the MO + H_2_O_2_ + Wor group)

*OS* the third passage of BMSCs were cultured in osteogenic differentiation medium, *MO* the third passage of BMSCs were cultured in osteogenic differentiation medium + 10% Moringa leaf-containing serum, *Wor* wortmannin, an inhibitor of PI3K, *DMSO* solvent of wortmannin

H_2_O_2_ treatment lasted 24 h starting at 0 h, and the cells in the MO + H_2_O_2_ + Wor group were treated with 0.5 μmol/L wortmannin for 24 h starting from 0 h. The OS medium or the medium supplemented with 10% Moringa leaf extract-containing serum was changed every 3 days. Cell cycle, oxidative stress, and protein expression were all assayed at 48 h after initiation of osteogenic differentiation. After 7 days of induction, the expression levels of genes for osteogenic markers were determined. ALP staining was examined on day 14, and osteogenesis determination was examined on day 21 after osteogenic differentiation.

### Preparation and analysis of drug-containing serum

Moringa leaf micropowder was provided by Heilongjiang Academy of Sciences (Harbin, China). Moringa leaf solution was made by dissolving the micropowder in saline and mixing well. Male rats weighing 250 ± 20 g were administered Moringa leaf solution through oral dosing (0.5 g/kg/day) at the frequency of twice per day for three consecutive days. Rats in the control group were given normal saline. Fifteen rats were used in preparation of drug-containing serum. At 1 h after the last dosing, blood was extracted from the heart after the euthanasia. Blood was collected and centrifuged at 300 rpm for 20 min at room temperature. The serum was sterilized by suction filtration and stored at – 20 °C. This study was approved by the Institutional Animal Care and Use Committee of Harbin Medical University.

The drug-containing serum was analyzed using Non-target High Performance Liquid Chromatography (HPLC). LC-MS/MS analyses were performed using an UHPLC system (1290, Agilent Technologies; Santa Clara, CA, USA) with a UPLC HSS T3 column (2.1 mm × 100 mm, 1.8 μm) coupled to Q Exactive (Orbitrap MS, Thermo Fisher Scientific; Waltham, MA, USA). The mobile phase A was 0.1% formic acid in water for positive, and 5 mmol/L ammonium acetate in water for negative, and the mobile phase B was acetonitrile. The elution gradient was set as follows: 0 min, 1% B; 1 min, 1% B; 8 min, 99% B; 10 min, 99% B; 10.1 min, 1% B; and 12 min, 1% B. The flow rate was 0.5 mL/min. The injection volume was 3 μL. The QE mass spectrometer was used because of its ability to acquire MS/MS spectra on an information-dependent basis (IDA) during an LC/MS experiment. In this mode, the acquisition software (Xcalibur 4.0.27, Thermo Fisher Scientific) continuously evaluates the full scan survey MS data as it collects and triggers the acquisition of MS/MS spectra depending on preselected criteria. ESI source conditions were set as follows: Sheath gas flow rate as 45 Arb, Aux gas flow rate as 15 Arb, capillary temperature 400 °C, full ms resolution as 70,000, MS/MS resolution as 17,500, collision energy as 20/40/60 eV in NCE model, and spray voltage as 4.0 kV (positive) or − 3.6 kV (negative), respectively.

The raw data were converted to the mzXML format using ProteoWizard and processed using MAPS software (Version 1.0). The preprocessing results generated a data matrix that consisted of the retention time (RT), massto-charge ratio (m/z) values, and peak intensity. In-house MS2 database was applied for metabolite identification.

### MTT assay

At 72 h after osteogenic differentiation under different treatment conditions, 20 μL MTT [3-(4,5-dimethylthiazol-2-yl)-2,5-diphenyltetrazolium bromide)] solution (5 g/L) was added to each well of the 96-well plates, and cells were incubated for another 4 h in the incubator (37 °C and 5% CO_2_). The medium in each well was aspirated and 100 μl of DMSO was added. Cell viability was quantified by measuring absorbance at 490 nm on a microplate reader. The optical density (OD) values of samples in experimental groups were normalized to that of the control groups without H_2_O_2_ or drug treatments at the same time point.

### Measurement of intracellular peroxide markers

The reactive oxygen species (ROS) test kit was purchased from Beyotime Biotechnology (Shanghai, China). The SOD (superoxide dismutase), MDA (malondialdehyde), and GSH-PX (glutathione peroxidase) test kits were obtained from Nanjing Institute of Bioengineering Research Institute (Nanjing, China). Assays to measure intracellular ROS and MDA levels, as well as SOD and GSH-PX activity of BMSCs were conducted following the manufacturer's protocols. Briefly, after removing the medium, 10 μmol/L DFCH-DA diluted in serum-free medium was added to each group of cells and incubated at 37 °C for 20 min in the dark. Then, the cells were washed three times with serum-free cell culture medium, and the fluorescence intensity of each well was detected using a fluorescence microplate reader at an excitation wavelength of 488 nm to calculate intracellular ROS levels. To determine SOD activity, total protein was extracted and the protein concentration was determined using the BCA method. The SOD inhibition rate was calculated according to the formula provided by the manufacturer. To determine MDA levels, the homogenate of treated cells was prepared, and the MDA value was calculated using a colorimetric measurement of each tube at 532 nm after the addition of the reagents. To detect GSH-PX activity, absorbance of the samples was determined at 412 nm at the end of the reaction using a microplate reader. All results were calculated according to the manuals provided with the kits.

### Cell cycle analysis

Cells were fixed with 70% ethanol at 4 °C overnight. After centrifugation at 1500 rpm for 5 min and two washes with PBS, cells were incubated with 0.1 mg/mL RNase A (Thermo Fisher Scientific) at 37 °C for 30 min and then 0.05 mg/ml propidium iodide (PI) at 4 °C for another 30 min. The DNA content was determined using a flow cytometer (FACSCalibur, BD Biosciences; Franklin Lakes, NJ, USA), and 2–3 million cells were collected. Data were analyzed using the Modfit LT software (Verity Software House).

### Osteogenesis-related gene expression

Total RNA was extracted using the Extreme RNA extraction kit (HaiGene; Harbin, China) following the manufacturer’s protocols. The quality of the total RNA was evaluated by the electrophoresis of the agarose gels, and the concentration of total RNA was measured using an ND5000 ultra-micro UV spectrophotometer. After that, the total RNA was reverse transcribed to cDNA using Golden 1st cDNA Synthesis Kit (HaiGene) followed the manufacturer's manual. In detail, 1μg of the total RNA was used in 20 μl of the reverse transcription reaction by mixing 4 μl of the 5× concentrated RT MasterMix (which contained the reaction buffer, MLV Reverse transcriptase, RNase Inhibitor, dNTPs, and Mg^2+^), 1 μl of 20× concentrated primer mixture of oligo dT and random hexamer primers, and distilled water. RNA was converted to cDNA following the reaction conditions: 25 °C for 5 min, 55 °C for 30 min, and 85 °C for 5 min. Then, 100 ng of the cDNA was used for quantitative real-time PCR (RT-qPCR) reaction on a fluorescence quantitative PCR system using SYBR Green Fluorescence Quantification PCR Kit (HaiGene). The reaction conditions were 15 min at 95 °C, 40 cycles of 10 s at 95 °C, and 30 s at 60 °C. The relative expression levels of each gene were analyzed using the 2^−ΔΔCt^ method and normalized to GAPDH expression. The sequences of primers for each gene were as follows:

ALP, 5′-CGGCGGATGATAAGGAGGAC-3′ (forward),

5′-GGCGTGTAACAGATGGAAACC-3′ (reverse);

Collagen I, 5′-AAGGGGTGGGGTGGGAAG-3′ (forward),

5′-GAGAGCAGGCTGGAGTTGG-3′ (reverse);

Osteopontin (OPN), 5′-GCTTGGCTTACGGACTGAGG-3′ (forward),

5′-CTGGGCAACTGGGATGACC-3′ (reverse);

Runx2, 5′-GCGGAACAACAACAACAACAAC-3′ (forward),

5′-GAAAGCAAATCTTGGGCAATAGC-3′ (reverse);

GAPDH, 5′-AACTCCCATTCTTCCACCTTT-3′ (forward),

5′-CTCTTGCTCTCAGTATCCTTG-3′ (reverse).

### Alkaline phosphatase (ALP) activity assays

ALP activity was detected using the ALP activity kit (Solarbio; Beijing, China) according to the manufacturer’s protocol. Briefly, BMSCs were fixed with the ALP fixative for 3 min. Then, the ALP incubation solution was added, and cells were incubated in the wet box for 20 min in dark. After washing with distilled water, the stained cells were visualized under a microscope and photographed.

### Quantifying matrix mineralization via alizarin red staining

Matrix mineralization was evaluated using alizarin red S staining. At 21 days after BMSC osteogenic differentiation under the indicated conditions, cells were washed twice with PBS and fixed with 4% paraformaldehyde for 30 min at 25 °C. Then, the cells were stained with 1% Alizalin Red (pH 4.2; Solarbio; Beijing, China) for 30 min and visualized under a microscope and photographed as previously described [30].

### Western blot analysis

Total protein was extracted from the treated cells with RIPA (radioimmunoprecipitation assay) lysis buffer and the total protein concentration was determined using a BCA assay kit (Beyotime; Shanghai, China). Protein samples in loading buffer were heated at 100 °C for 5 min, and an equal amount of protein for each sample was loaded onto 12% polyacrylamide gels (Beyotime Biotechnology). After electrophoresis, proteins were transferred onto polyvinylidene difluoride membranes. The membranes were blocked with 5% BSA and incubated with the primary antibodies overnight a 4 °C and the secondary antibody for 1 h at room temperature. The primary antibodies were as follows: anti-caspase-3 (1:1,000 dilution; Cell Signaling Technology; Danvers, MA, USA), anti-Akt (1:1,000 dilution; Cell Signaling Technology), anti-phosphorylated (p)-Akt (1:2,000 dilution; Cell Signaling Technology), anti-Foxo1 (1:1,000 dilution; Cell Signaling Technology), and anti-GAPDH (Zhongshan Golden Bridge Biotechnology Co., Ltd., Beijing, China). Antibodies were diluted in Tris-buffered saline with 0.1% Tween-20 (pH 7.6, 20 mM Tris and 150 mM NaCl; TBS-T). Secondary antibodies including goat anti-mouse (H+L)/HRP (horseradish peroxidase) (1:5,000 dilution) and goat anti-mouse (H+L)/HRP (1:5,000 dilution) were purchased from Zhongshan Golden Bridge Biotechnology Co., Ltd. (Beijing, China). Proteins of interest were visualized using the Supersensitive ECL (enhanced chemiluminescence) Primer kit purchased from HaiGene (Harbin, China). Signals were detected using an Imaging System (Clinx; Shanghai, China) and data are expressed as normalized ratios to GAPDH.

### Statistical analysis

**Fig. 1:**
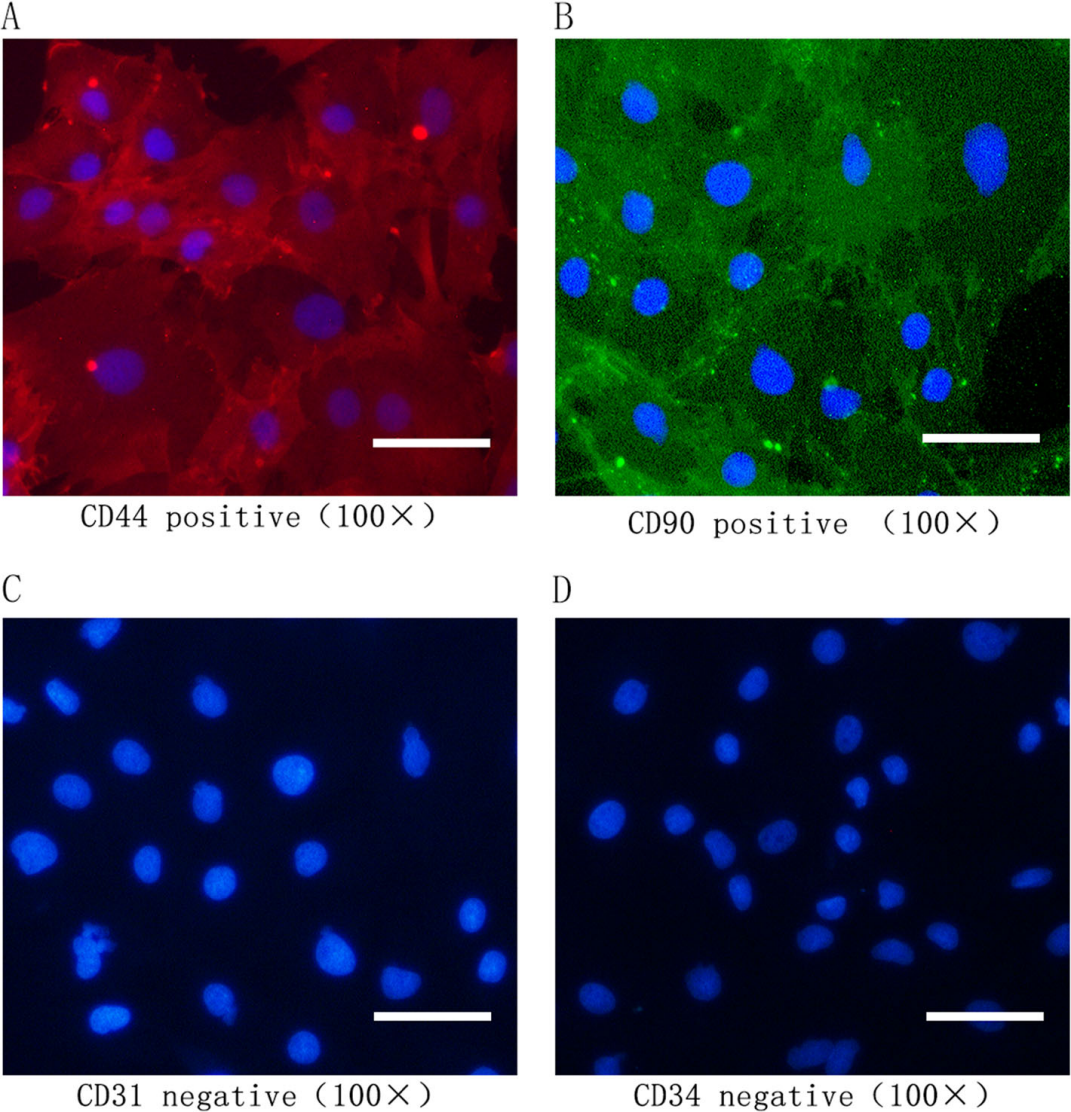
*Moringa oleifera* attenuates H_2_O_2_-induced dysregulation of cell viability and cell cycle progression in BMSCs during osteogenic differentiation. **a** The effects of H_2_O_2_ at indicated concentrations and treatment times on cell viability in P3 cells. H_2_O_2_50, cells treated with 50 μmol/L H_2_O_2_; H_2_O_2_100, cells treated with 100 μmol/L H_2_O_2_; H_2_O_2_200, cells treated with 200 μmol/L H_2_O_2_. *P < 0.05 compared with the control group; ^#^*P* < 0.05 compared with the H_2_O_2_50 group; ^@^*P* < 0.05 compared with the H_2_O_2_100 group. **b**, **c** The effects of H_2_O_2_ and *Moringa oleifera* on cell viability and cell cycle progression of BMSCs during osteogenic differentiation. Cell viability (**b**) and cell cycle progression (**c**) of the indicated groups were measured at 48 h after initiation of osteogenic differentiation. Representative flow profile of cell cycle progression and percentage of cells in the G2 + S phases are shown. *N* = 3 for each group; *P < 0.05 compared with the control group; ^#^*P* < 0.05 compared with the MO group; ^@^*P* < 0.05 compared with the OS + H_2_O_2_ group

**Fig. 2:**
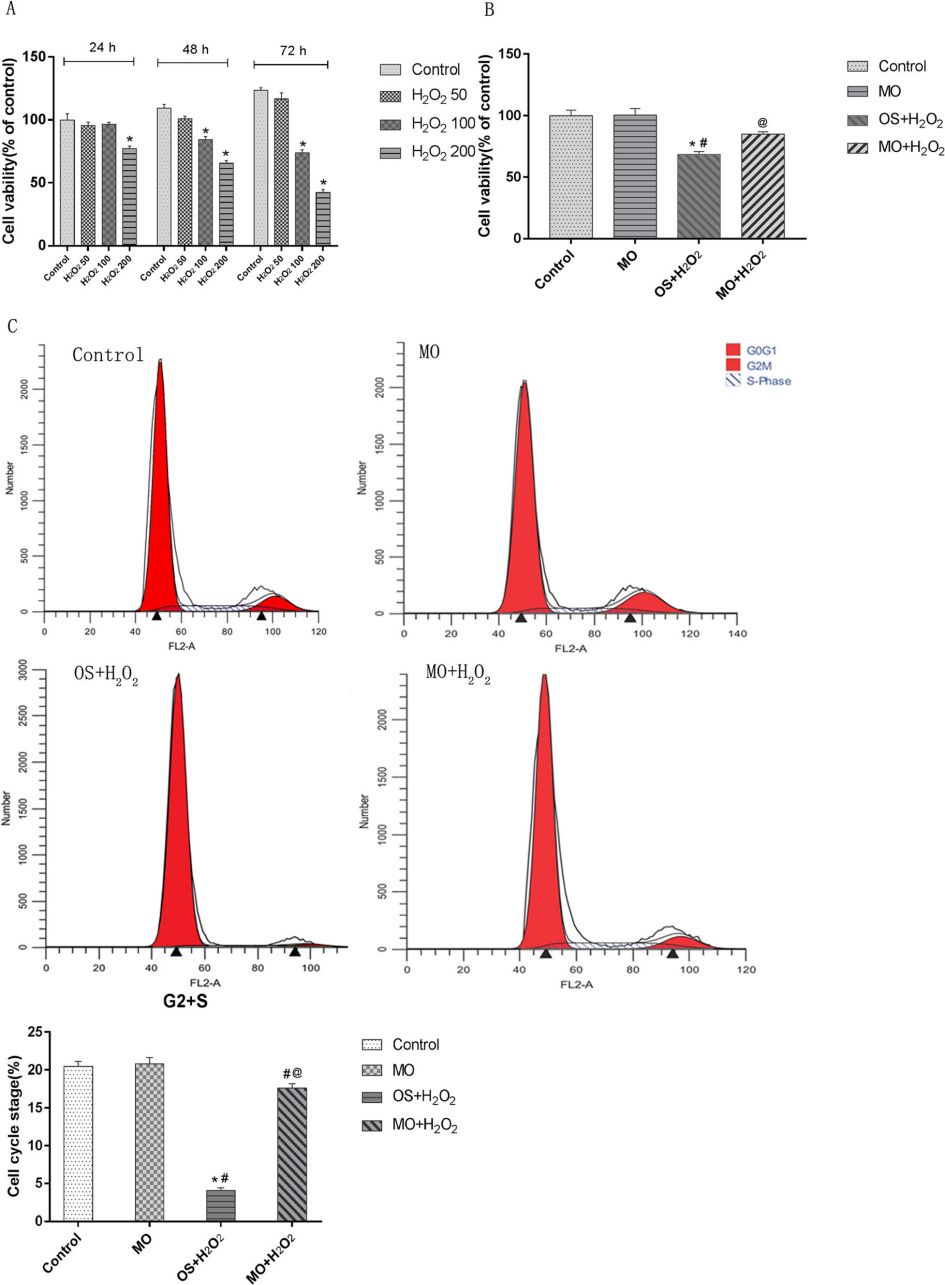
The effects of H_2_O_2_ and *Moringa oleifera* on intracellular peroxide markers during osteogenic differentiation. **a**–**d** At 48 h after initiation of osteogenic differentiation, ROS levels (**a**), MDA levels (**b**), SOD activity (**c**), and GSH-PX activity (**d**) of BMSCs under the indicated osteogenic induction conditions were measured. *N* = 3 for each group; *P < 0.05 compared with the control group; ^#^*P* < 0.05 compared with the MO group; ^@^*P* < 0.05 compared with the OS + H_2_O_2_ group

**Fig. 3:**
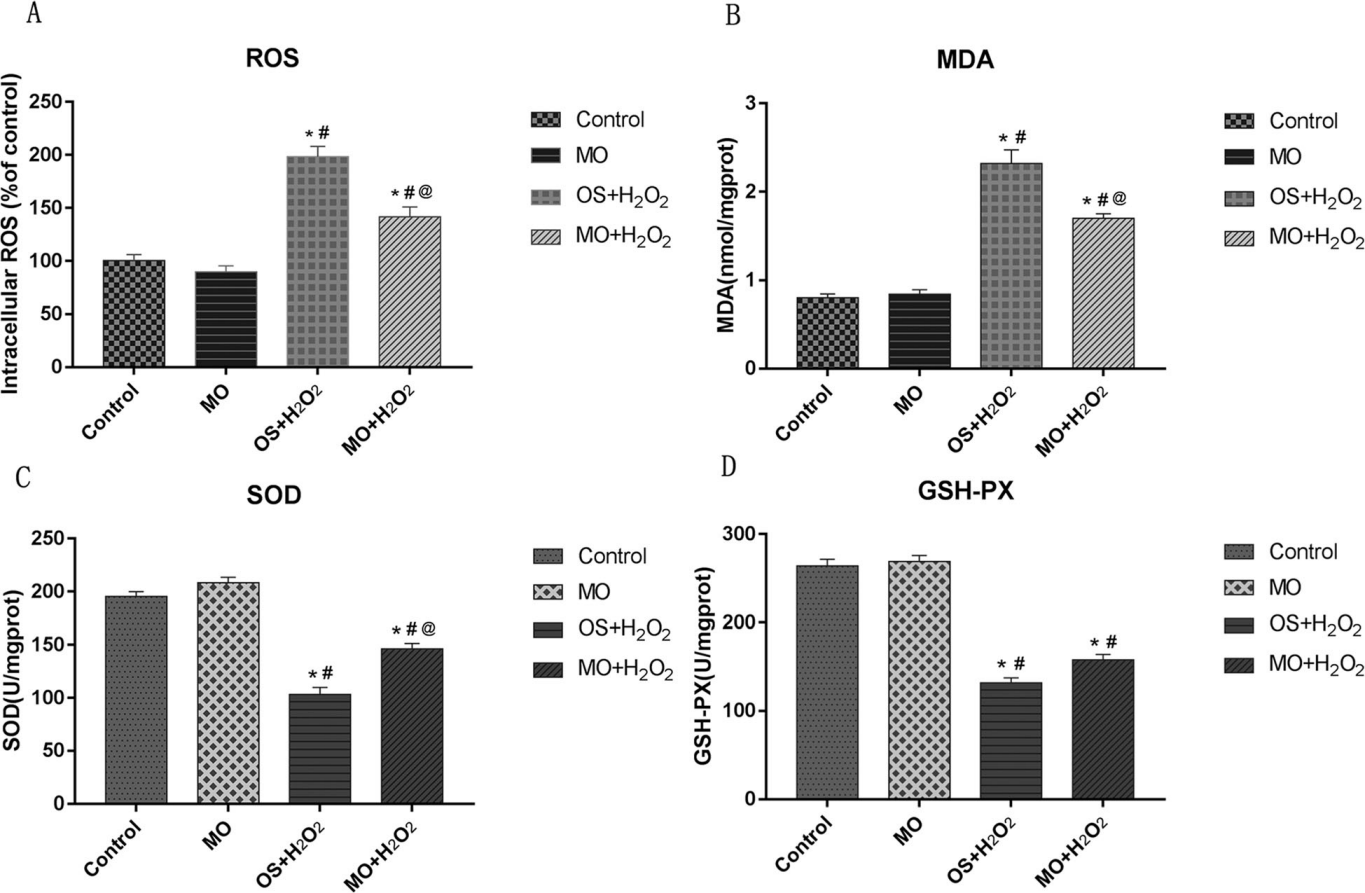
The effects of H_2_O_2_ and *Moringa oleifera* on expression of Foxo1 and cleaved caspase-3 and pAkt/Akt in BMSCs during osteogenic differentiation. **a**, **b** At 48 h after initiation of osteogenic differentiation, protein expression of Foxo1, Akt, and pAkt in BMSCs under the indicated osteogenic induction conditions were measured using western blot assays. **a** *n* = 3 for each group; *P < 0.05 compared with the control group; ^#^*P* < 0.05 compared with the MO group; ^@^*P* < 0.05 compared with the OS + H_2_O_2_ group. **b** *n* = 3 for each group; *P < 0.05 compared with the MO + H_2_O_2_ + DMSO group, ^#^*P* < 0.05 compared with the MO + H_2_O_2_ group. **c** Protein expression of cleaved caspase-3 in BMSCs treated with 100 μmol/L H_2_O_2_ for the indicated duration was measured using western blot assay. *n* = 3 for each group; *P < 0.05 compared with the control group. **d** At 48 h after initiation of osteogenic differentiation, protein expression of cleaved caspase-3 in BMSCs under the indicated osteogenic induction conditions was measured using western blot assays. *N* = 3 for each group; *P < 0.05 compared with the MO group; ^#^*P* < 0.05 compared with the OS + H_2_O_2_ group; ^@^*P* < 0.05 compared with the MO + H_2_O_2_ group

**Fig. 4:**
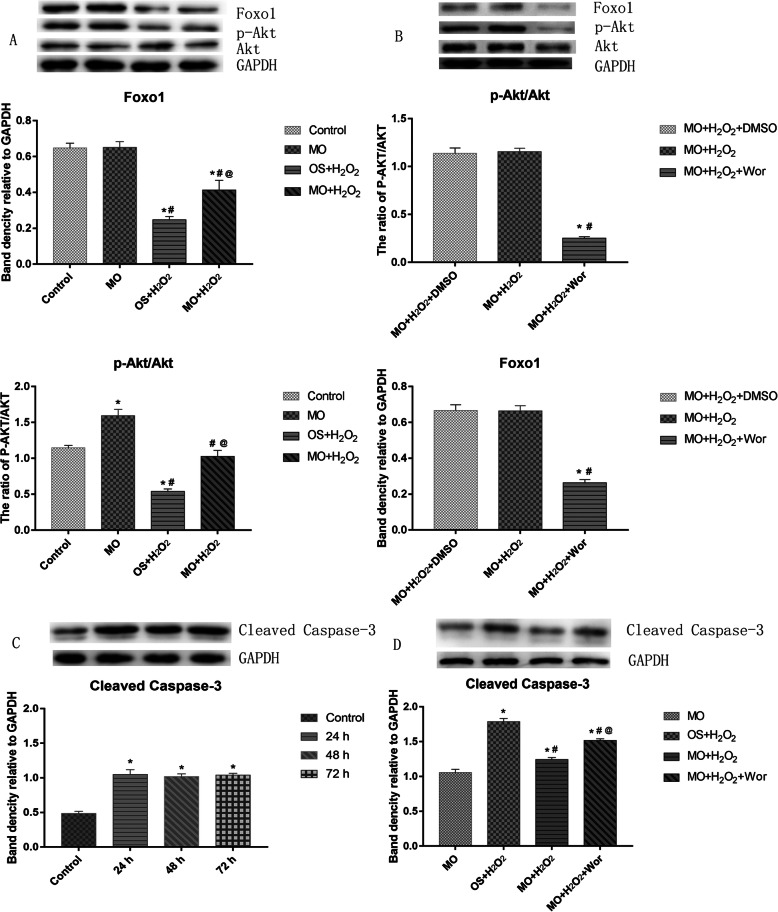
The effect of H_2_O_2_ and *Moringa oleifera* on expression of osteogenic markers in BMSCs during osteogenic differentiation. **a A**t 7 days after initiation of osteogenic differentiation, mRNA levels of ALP, collagen I, OPN, and Runx2 in BMSCs under the indicated osteogenic induction conditions were measured using qRT-PCR. *N* = 3 for each group; *P < 0.05 compared with the control group; ^#^*P* < 0.05 compared with the MO group; ^@^*P* < 0.05 compared with the OS + H_2_O_2_ group; ^&^*P* < 0.05 compared with the MO + H_2_O_2_ group. **b** Representative images of ALP staining at 7 days and alizarin red staining at 21 days after the initiation of osteogenic differentiation in BMSCs. Magnification, × 40; scale bar, 200 μm

The experimental results are expressed as mean ± standard deviation (mean ± SD), and statistical analysis was performed using the SPSS 24.0 software (IBM, Armonk, NY, USA). One-way analysis of variance (one-way ANOVA) was used to compare multiple-sample groups. Differences between groups were analyzed by Tukey’s post hoc test (Figs. 1, 2, 3a, c, d, and 4) or Dunnett’s T3 test (Fig. 3b). Differences were statistically significant at *P* < 0.05.

## Results

### The effects of H_2_O_2_ and Moringa oleifera on cell viability and cell cycle progression of BMSCs during osteogenic differentiation

**Fig. 5:**
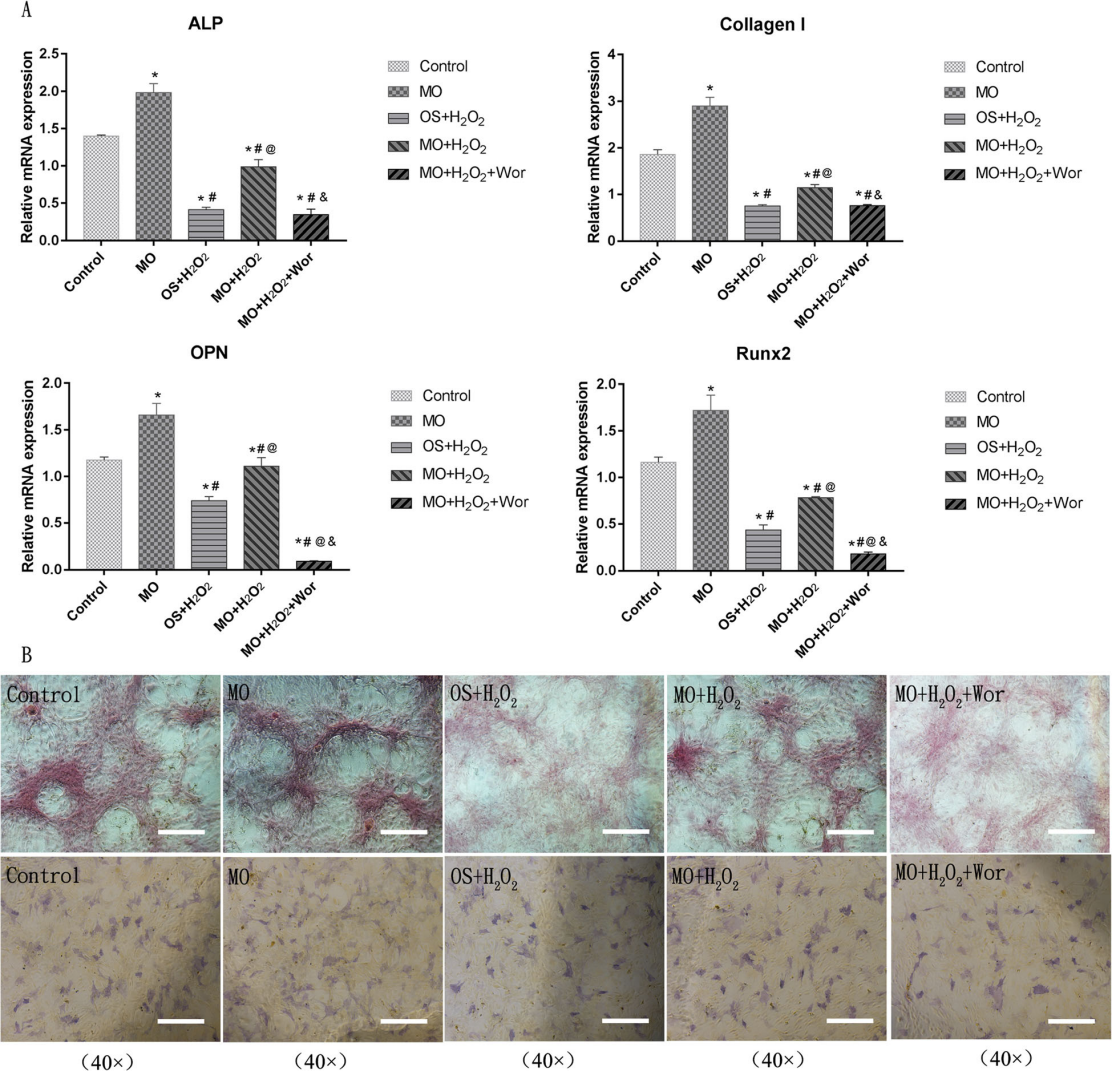
Characterization of BMSCs with immunofluorescence staining. The expression levels of CD44, CD90, CD31, and CD34 in the differentiated cells were characterized by indirect immunofluorescence assays using primary monoclonal antibodies against the indicated antigens and fluorescent secondary antibodies. The data are representative images with the indicated magnifications obtained from three independent experiments. **a** CD44 expression in P3 BMSCs. **b** CD90 expression in P3 BMSCs. **c** Negative CD31 expression in BMSCs. **d** Negative CD34 expression in BMSCs. Scale bar, 100 μm

The P3 cells (3rd generation of primary rat BMSCs) with good growth status were used for in vitro experiments. These BMSCs were confirmed to be CD44^+^ CD90^+^ CD31^−^ CD34^−^ using immunofluorescence antibody staining (Fig. 5a–d). To test the therapeutic effects of *Moringa oleifera* during BMSC osteogenic differentiation, we first sought to establish a BMSCs damage model. The P3 cells were treated with H_2_O_2_ at the concentrations of 50 μmol/L, 100 μmol/L, and 200 μmol/L for 24, 48, and 72 h, separately. Cell viability was measured to evaluate cellular damage. As shown in Fig. 1a, no significant difference was observed in cell viability of BMSCs treated with 50 μmol/L and 100 μmol/L doses of H_2_O_2_ for 24 h or 50 μmol/L H_2_O_2_ for 48 h and 72 h. Cell viability of BMSCs treated for 48 h significantly decreased upon H_2_O_2_ challenge, and further decreased in response to 200 μmol/L H_2_O_2_ (*P*_200 VS Control_ < 0.001). Cell viability decreased in BMSCs treated with 100 μmol/L H_2_O_2_ for 48 h (*P*_100 VS Control_ = 0.006). According to the above results, BMSCs treated with 100 μmol/L H_2_O_2_ were selected for subsequent experiments, and the P3 cells were treated for either 48 h, 7 days, or 21 days.

**Fig. 6:**
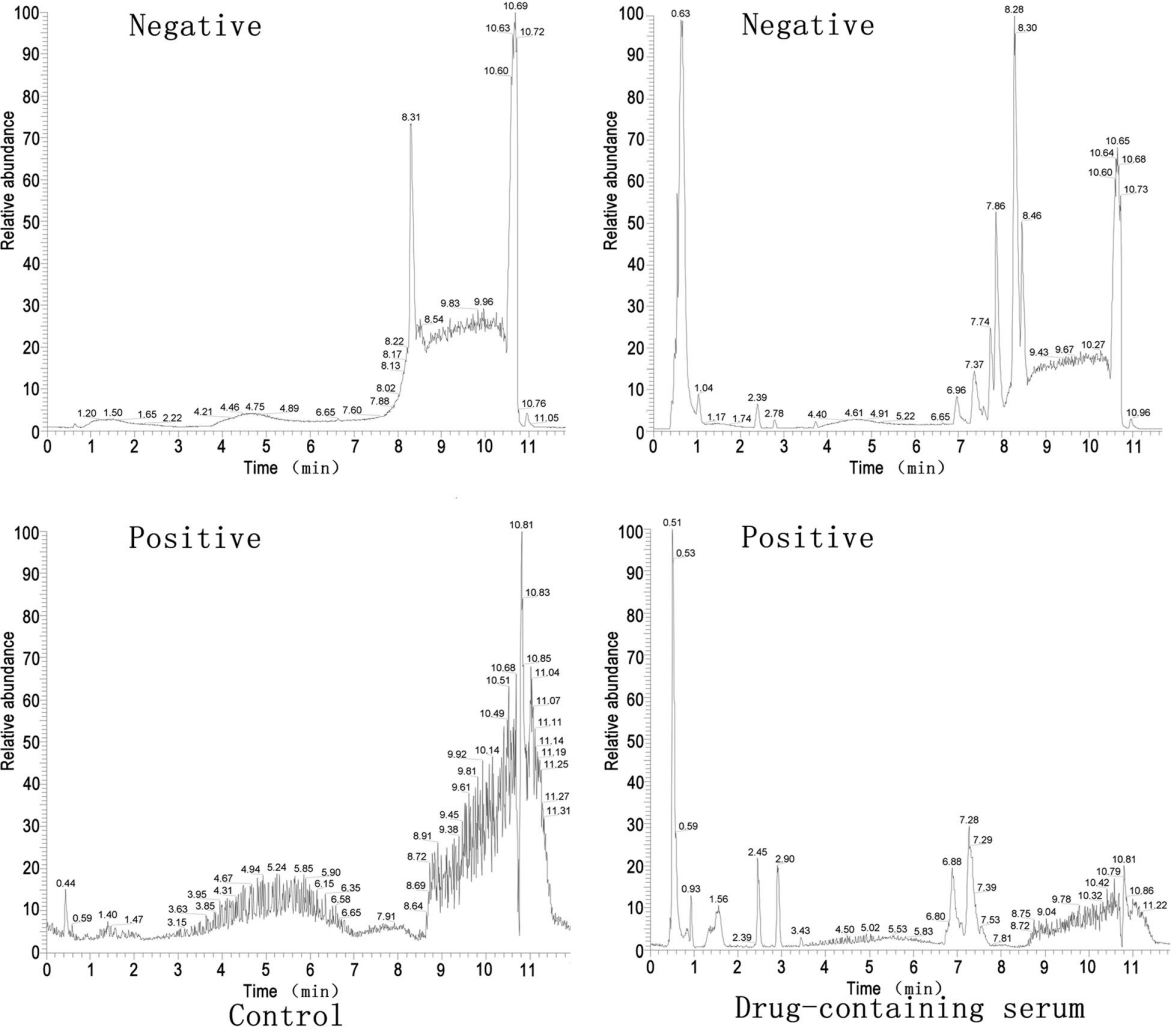
Non-target UHPLC-QE-MS analysis confirmed the existence of *Moringa oleifera* in the serum of rats that received oral dosing of Moringa leaf solution. HPLC analysis shows the differences in drug-containing serum from rats administrated Moringa leaf solution for three consecutive days (right panels) and blank serum components from rats administrated control saline (left panels). The differences in components between the two sets of samples are significant in both the negative ion mode (upper panels) and the positive ion mode (lower panels), as indicated by the different patterns of peaks. Further targeted analyses are needed to identify what ingredients the specific peaks are

We next evaluated the impact of *Moringa oleifera* on attenuating dysregulated cell viability and cell cycle progression of BMSCs treated with 100 μmol/L H_2_O_2_. To better mimic the clinical pharmacodynamic property of *Moringa oleifera* in patients, we treated BMSCs with drug-containing serum from rats administrated the Moringa leaf for three consecutive days. The non-target UHPLC-QE-MS analysis confirmed the existence of *Moringa oleifera* in the serum (Fig. 6). After treatment for 48 h, BMSCs demonstrated significant differences in cell viability and proliferation (as indicated by the percentage of cells in the G2 + S phases of cell cycle progression) among the control, MO, OS + H_2_O_2_, and MO + H_2_O_2_ groups. Cell viability (Fig. 1b) and proliferation (Fig. 1c) in the control and MO groups were significantly higher compared to the OS + H_2_O_2_ groups (all *P* ≤ 0.05). Compared to the control group, cell viability and proliferation were not altered in the MO group. Notably, compared to the OS + H_2_O_2_ group, cell viability and proliferation in the MO + H_2_O_2_ group improved significantly (all *P* ≤ 0.05), suggesting that *Moringa oleifera* can significantly reduce H_2_O_2-_mediated damage of BMSCs.

### The effect of H_2_O_2_ and Moringa oleifera on intracellular peroxidative markers of BMSC osteogenic differentiation

Next, we evaluated the effects of H_2_O_2_ and *Moringa oleifera* on BMSC intracellular peroxide markers, including ROS and MDA levels, as well as SOD and GSH-PX activity. During BMSC osteogenic differentiation, significant differences were identified in these parameters among the four groups (Fig. 2). ROS (Fig. 2a) and MDA (Fig. 2b) levels in the control and MO groups were significantly lower compared to the OS + H_2_O_2_ and MO + H_2_O_2_ groups (all *P* ≤ 0.001), while SOD (Fig. 2c) and GSH-PX (Fig. 2d) activity in the OS + H_2_O_2_ and MO + H_2_O_2_ groups were significantly lower compared to the Control and MO groups (all *P* ≤ 0.001).

No significant differences were found in ROS and MDA levels, as well as SOD and GSH-PX activity between the control and MO groups. ROS and MDA levels in the MO + H_2_O_2_ groups were significantly lower compared to the OS + H_2_O_2_ group (all *P* < 0.05). In contrast, SOD and GSH-PX activity in the MO + H_2_O_2_ groups were significantly higher compared to the OS + H_2_O_2_ group (all *P* < 0.05), except *P*_GSH-PX_ = 0.099. Collectively, these results suggest that *Moringa oleifera* can significantly alleviate the intracellular H_2_O_2_-mediated perturbations in BMSCs.

### The effects of H_2_O_2_ and Moringa oleifera on Foxo1 and cleaved caspase-3 expression and the ratio of pAkt/Akt in BMSCs during osteogenic differentiation

Since the PI3K/Akt signaling pathway is critically involved in BMSC osteogenic differentiation [31], we evaluated the effects of H_2_O_2_ and *Moringa oleifera* in regulating the expression levels of Foxo1 and pAkt, two downstream targets of the PI3K/Akt pathway. There were significant differences in the expression levels of Foxo1 and the ratios of pAkt/Akt among the four groups (Fig. 3a). The ratio of pAkt/Akt in the MO group was significantly higher compared to the other groups (all *P* < 0.05, Fig. 3a), while the expression of Foxo1 and the pAkt/Akt ratio were lowest in the OS + H_2_O_2_ group (all *P* < 0.05, Fig. 3a). No significant differences were found in the pAkt/Akt ratio between the control group and the MO + H_2_O_2_ group, or in Foxo1 expression between the control group and the MO group (Fig. 3a).

To substantiate the role of PI3K/Akt signaling in the protective effect of *Moringa oleifera*, we treated BMSCs in the MO + H_2_O_2_ group with the vehicle DMSO or the PI3K inhibitor wortmannin. As shown in Fig. 3b, DMSO had no effect on Akt and Foxo1 expression, while the pAkt/Akt ratio and Foxo1 expression in the MO + H_2_O_2_ + Wor group were significantly lower compared to the MO + H_2_O_2_ or MO + H_2_O_2_ + DMSO group (all *P* < 0.001).

PI3K/Akt signaling also plays an important role in survival pathways by inactivating downstream apoptotic factors. Therefore, we further examined whether the protective effect of *Moringa oleifera* was associated with reduced apoptosis in BMSCs stimulated with H_2_O_2_. First, we detected expression of cleaved caspase-3 in BMSCs treated with 100 μmol/L H_2_O_2_ for different durations. As shown in Fig. 3c, H_2_O_2_ at all tested durations (24, 48, and 72 h) induced significantly higher expression of cleaved caspase-3 compared to the control group (Fig. 3c). However, no significant difference was found among the groups with different treatment durations.

We then examined the impact of H_2_O_2_, *Moringa oleifera*, and wortmannin, alone or in combination, on cleaved caspase-3 expression in BMSCs during osteogenic differentiation. As shown in Fig. 3d, there were significant differences in the expression of cleaved caspase-3 among different treatment groups. Cleaved caspase-3 expression was highest in the OS + H_2_O_2_ group (*P* < 0.05), and lowest in the MO group (all *P* < 0.05). Compared to the MO + H_2_O_2_ group, cleaved caspase-3 expression was higher in the OS + H_2_O_2_ group and MO + H_2_O_2_ + Wor group (all *P* < 0.05). Taken together, these results indicate that *Moringa oleifera* exerts protective effects upon H_2_O_2_-induced damage of BMSCs through enhancing PI3K/Akt signaling and reducing BMSC apoptosis.

### The effect of H_2_O_2_ and Moringa oleifera on the expression of osteogenic markers in BMSCs during osteogenic differentiation

We also evaluated the impact of H_2_O_2_, *Moringa oleifera*, and wortmannin on the expression levels of osteogenic genes (ALP, collagen I, OPN, and Runx2) in BMSCs. As shown in Fig. 4a, after 7 days of treatment, there were significant differences in gene expression among the control, MO, OS + H_2_O_2_, MO + H_2_O_2_, and MO + H_2_O_2_ + Wor groups. Expression levels of ALP, collagen I, and Runx2 in the Control group were higher compared to the OS + H_2_O_2_ and MO + H_2_O_2_ group (all *P* < 0.05). Compared to the control group, OPN expression in the OS + H_2_O_2_ group decreased significantly (*P* = 0.007), while additional *Moringa oleifera* treatment (MO + H_2_O_2_ group) significantly reversed this decrease (*P* < 0.05).

The expression levels of four osteogenic markers in the MO + H_2_O_2_ group were higher compared to those in the OS+H_2_O_2_ group. No evident changes in ALP, collagen I, or Runx2 expression were identified between the OS + H_2_O_2_ and the OS + H_2_O_2_ + Wor groups, suggesting that wortmannin can eliminate the protective effects of *Moringa oleifera* in terms of promoting osteogenic gene expression. In addition, compared to the OS + H_2_O_2_ + Wor group, expression levels of ALP, collagen I, OPN, and Runx2 in the control, MO, and MO + H_2_O_2_ groups were significantly higher (all *P* < 0.05). Expression levels of ALP, collagen I, OPN, and Runx2 in the MO group were significantly higher compared to all other groups (all *P* < 0.05).

Furthermore, the improvement in BMSC osteogenic differentiation by *Moringa oleifera* was confirmed through ALP staining and alizarin red staining in the above mentioned five groups. ALP staining was performed after culturing the P3 cells for 7 days. The number of blue stained cells in the MO + H_2_O_2_ group was higher compared to the OS + H_2_O_2_ group, while the number of positively stained cells in the MO group was the highest. Wortmannin significantly reduced the number of positively stained cells cultured in the medium with MO + H_2_O_2_ (Fig. 4b). Alizarin red staining was carried out at 21 days after initiation of osteogenic differentiation, and the results were consistent with the ALP staining (Fig. 4b).

## Discussion

Cell peroxidation is an important factor in aging and various chronic diseases. Research in the last 20 years has revealed that oxidative stress plays central roles in the etiology of osteoporosis [32]. In this study, we established a model of peroxidative damage in rat BMSCs undergoing osteogenic differentiation and evaluated the protective roles of *Moringa oleifera* in rescuing H_2_O_2_-mediated changes in BMSC cell viability, cell cycle progression, oxidative stress, and expression of osteogenic markers. *Moringa oleifera* leaf exacts-containing serum significantly increased the expression of osteogenic differentiation associated genes, increased ALP activity and calcium deposition, maintained cell viability and proliferation, and relieved oxidative stress caused by H_2_O_2_ in BMSCs. Further mechanistic studies suggested that the protective roles of *Moringa oleifera* in damaged BMSCs were associated with the activation of the PI3K/Akt signaling pathway and reduced apoptosis.

ROS can activate caspase-3, which in turn triggers the caspase cascade leading to apoptosis [33]. In this study, cell viability and proliferation of H_2_O_2_-treated BMSCs decreased significantly at 48 h after treatments, while intracellular ROS and MDA levels increased significantly, and SOD and GSH-PX activity decreased. Consistently, levels of cleaved caspase-3, a marker of apoptosis, increased significantly in the H_2_O_2_-treated BMSCs starting at 24 h after treatments. These results indicate that H_2_O_2_ can significantly reduce cell viability and proliferation by activating intracellular oxidative stress and apoptosis. In addition, in vitro peroxidative damage has also been reported to exert a negative effect on osteogenic differentiation [34, 35]. We found that H_2_O_2_ not only decreased cell viability and the cell proliferation index in BMSCs, but also significantly reduced expression of osteogenic differentiation-associated genes, including ALP, collagen I, OPN, and Runx2. *Moringa oleifera* leaf extracts-containing serum partially reversed the H_2_O_2_-induced changes, and this effect was blocked by wortmannin. Moreover, the therapeutic effects of *Moringa oleifera* in enhancing BMSC osteogenic differentiation were further confirmed by ALP and alizarin red staining, an important marker of cellular calcium deposition [36]. However, *Moringa oleifera* leaf extracts-containing serum did not change these indicators under normal culture conditions.

Our results suggest that *Moringa oleifera* leaf exacts-containing serum activates PI3K, which plays a role in promoting osteogenic differentiation of H_2_O_2_-damaged BMSCs. In mouse bone pre-osteoblast MC3T3-E1 cells, the PI3K/Akt signaling pathway was also shown to promote osteogenic differentiation [37]. Studies have shown that activation of Akt enhances the activity of the transcription factor MyoD in myoblasts, thus inducing differentiation into myocytes and incorporation into regenerative muscle fibers [38–40]. Here, we showed that the Akt pathway was activated during BMSC osteogenic induction. *Moringa oleifera* leaf extracts-containing serum further increased the ratio of pAkt/Akt and promoted osteogenic induction. Akt activation was significantly decreased in H_2_O_2_ injured cells, while *Moringa oleifera* leaf exacts-containing serum prevented the decrease in the pAkt/Akt ratio caused by H_2_O_2_. It is worth noting that *Moringa oleifera* leaf exacts-containing serum also increased the ratio of pAkt/Akt in BMSCs without H_2_O_2_ challenge. The detailed mechanisms by which *Moringa oleifera* leaf exacts enhance PI3K/Akt signaling under stress and non-stress conditions need to be further investigated.

Foxo1 acts as an important regulator of antioxidant stress and oxygen free radical balance during bone formation. The PI3K/Akt/Foxo1 pathway has been reported to play an important role in bones and muscles [41–44]. However, recent studies have shown that the role of Foxo1 in osteogenic differentiation is controversial. It has been reported that Foxo1 expression was increased after inhibition of PI3K [44], while another study reported that FOXO deletion in Osx1-Cre-expressing cells increased bone formation by activating Wnt signaling [45]. Most studies suggest that Foxo1 can improve osteogenic differentiation of osteoblasts. For example, Foxo1 gene knockout mice have reduced bone density and reduced osteogenic differentiation [46]. It has been reported that suppressing Foxo1 expression inhibits osteoblast proliferation, inhibits bone formation, reduces bone density, and increases oxidative stress levels [47]. Furthermore, it been shown that TNF-α inhibits Foxo1 by up-regulating miR-705, aggravating oxidative damage in BMSCs during osteoporosis [48]. In this study, we confirmed that oxidative damage by H_2_O_2_ decreased Foxo1, which was reversed by *Moringa oleifera* leaf extracts-containing serum. Therefore, our study suggests that Foxo1 has an important role in attenuating oxidative damage in BMSCs undergoing osteogenic differentiation. Under normal culture conditions, *Moringa oleifera* leaf extracts-containing serum did not change Foxo1 expression in BMSCs. In contrast, Foxo1 expression showed a trend consistent with that of Akt phosphorylation in response to H_2_O_2_ stimulation, and this trend was reversed by wortmannin. These results indicate that PI3K/Akt/Foxo1 signaling in BMSC osteogenic differentiation might be augmented in response to peroxidative damage.

This study has some limitations. First, the *Moringa oleifera* leaf contained a variety of active ingredients [49]. In this study, we only performed non-target analysis of drug-containing serum, which was based on a preliminary experiment. Although we did demonstrate that the drug-containing serum was effective, further analyses of specific active ingredients in the serum with assays like a physicochemical analysis will require additional substantial work, and we believe this can help understand the more in-depth cellular and molecular mechanisms of the *Moringa oleifera* leaf in anti-oxidation and modulating osteogenic differentiation of BMSCs. Second, in the present study, rat BMSCs were isolated and confirmed with a typical morphology. They were positive for CD44 and CD90 and were negative for CD31 and CD34. However, we did not have a cell line with a known surface marker expression pattern as a positive control to authenticate MSCs. Third, we used rat serum and fetal bovine serum in our experiments, and found no statistical difference in the tested indicators between these two serum treatments (data not shown). For ethical considerations, rat serum was used for the experiments involving drug-containing serum, while the remaining experiments were performed with culture medium supplemented with fetal bovine serum.

In summary, our results show that H_2_O_2_ can negatively regulate osteogenic induction of rat BMSCs through inhibiting cell proliferation, reducing cell viability, causing oxidative damage, and inactivating the PI3K/Akt/Foxo1 pathway. *Moringa oleifera* leaf extracts-containing serum positively regulated BMSC osteogenic differentiation through restoring cell proliferation and viability, reducing peroxidative damage, inhibiting apoptosis, and activating the PI3K/Akt/Foxo1 pathway. Our work supports the clinical use of *Moringa oleifera* leaf extracts to treat patients with osteoporosis due to aging and peroxidative damage, and also suggests that administration of *Moringa oleifera* leaf extracts could be a promising strategy to prevent osteoporosis and reduce frailty.

## Data Availability

The analyzed datasets generated during the study are available from the corresponding author on reasonable request.

## Abbreviations

AKT: Protein kinase B
BMSC: Bone marrow mesenchymal stem cell
DMEM: Dulbecco’s modified Eagle’s medium
FoxO1: Forkhead Box O1
GSH-PX: Glutathione peroxidase
H2O2: Hydrogen peroxide
MDA: Malondialdehyde
MTT: 3-(4,5-dimethylthiazol-2-yl)-2,5-diphenyl-2-H-tetrazolium bromide
OS: Third passage of BMSCs was cultured in osteogenic differentiation medium
MO: Third passage of BMSCs was cultured in osteogenic differentiation medium supplemented with 10% Moringa leaf extract-containing serum
P3: Third passage of BMSCs
PI3K: Phosphatidylinositol 3-kinase
ROS: Reactive oxygen species
SEM: Standard error of the mean
SOD: Superoxide dismutase
TBST: Tris-buffered saline Tween 20
Wor: Wortmannin
